# Rapid Large-Scale COVID-19 Testing during Shortages

**DOI:** 10.3390/diagnostics10070464

**Published:** 2020-07-08

**Authors:** Christian Beetz, Volha Skrahina, Toni M. Förster, Hanaa Gaber, Jefri J. Paul, Filipa Curado, Arndt Rolfs, Peter Bauer, Stephan Schäfer, Volkmar Weckesser, Vivi Lieu, Mandy Radefeldt, Claudia Pöppel, Susann Krake, Krishna K. Kandaswamy, Katja Bruesehafer, Florian Vogel

**Affiliations:** 1 CENTOGENE AG, 18055 Rostock, Germany; volha.skrahina@centogene.com (V.S.); Toni.Foerster@centogene.com (T.M.F.); Hanaa.Gaber@centogene.com (H.G.); Jefri.Paul@centogene.com (J.J.P.); Filipa.Curado@centogene.com (F.C.); Arndt.Rolfs@centogene.com (A.R.); Peter.Bauer@centogene.com (P.B.); Volkmar.Weckesser@centogene.com (V.W.); Vivi.Lieu@centogene.com (V.L.); Mandy.Radefeldt@centogene.com (M.R.); Claudia.Poeppel@centogene.com (C.P.); Susann.Krake@centogene.com (S.K.); Krishna.Kandaswamy@centogene.com (K.K.K.); Katja.Bruesehafer@centogene.com (K.B.); Florian.Vogel@centogene.com (F.V.); 2 Medizinisches Versorgungszentrum Labor Limbach Vorpommern Rügen, 18435 Stralsund, Germany; stephanxschaefer@web.de

**Keywords:** COVID-19, preventive testing, RT-PCR, SARS-CoV-2, testing, validation

## Abstract

The Coronavirus disease 2019 (COVID-19) pandemic caused by the Severe Acute Respiratory Syndrome Coronavirus-2 (SARS-CoV-2) has resulted in economic and social lockdowns in most countries all over the globe. Early identification of infected individuals is regarded as one of the most important prerequisites for fighting the pandemic and for returning to a ‘New Normal’. Large-scale testing is therefore crucial, but is facing several challenges including shortage of sample collection tools and of molecular biological reagents, and the need for safe electronic communication of medical reports. We present the successful establishment of a holistic SARS-CoV-2 testing platform that covers proband registration, sample collection and shipment, sample testing, and report issuing. The RT-PCR-based virus detection, being central to the platform, was extensively validated: sensitivity and specificity were defined as 96.8% and 100%, respectively; intra-run and inter-run precision were <3%. A novel type of sample swab and an in-house-developed RNA extraction system were shown to perform as good as commercially available products. The resulting flexibility guarantees independence from the current bottlenecks in SARS-CoV-2 testing. Based on our technology, we offered testing at local, national, and global levels. In the present study, we report the results from approx. 18,000 SARS-CoV-2 tests in almost 10,000 individuals from a low-frequency SARS-CoV-2 pandemic area in a homogenous geographical region in north-eastern Germany for a period of 10 weeks (21 March to 31 May 2020). Among the probands, five SARS-CoV-2 positive cases were identified. Comparative analysis of corresponding virus genomes revealed a diverse origin from three of the five currently recognized SARS-CoV-2 phylogenetic clades. Our study exemplifies how preventive SARS-CoV-2 testing can be set up in a rapid and flexible manner. The application of our test has enabled a safe maintenance/resume of critical local infrastructure, e.g., nursing homes where more than 5000 elderlies and caretakers got tested. The strategy outlined by the present study may serve as a blueprint for the implementation of large-scale preventive SARS-CoV-2 testing elsewhere.

## 1. Introduction

Corona Virus Disease 2019 (COVID-19) is caused by a novel coronavirus named Severe Acute Respiratory Syndrome Coronavirus 2 (SARS-CoV-2) [1]. In December 2019, the first infections were reported from Wuhan (China), and COVID-19 was declared a pandemic by the World Health Organization (WHO) on 11 March 2020. Health officials soon considered rapid and widespread testing an appropriate measure to fight the outbreak [2].

With the respiratory tract being the primary body entry site for SARS-CoV-2 [3], bronchoalveolar fluid, as well as nasopharyngeal and oropharyngeal samples, have been suggested for detection of acute infection [4,5]. The latter two are amenable to outpatient sampling, but oropharyngeal sampling is less discomforting and easier to perform, including in a self-sampling setting [6]. Wet pharyngeal sampling (i.e., usage of a transport medium) and dry pharyngeal sampling (no medium involved) are generally considered to perform similarly well [7].

For detecting SARS-CoV-2 in proband samples, reverse transcription quantitative polymerase chain reaction (RT-qPCR) following a WHO-recommended approach [8] has usually been applied. Several modifications regarding the RNA extraction step (e.g., [9]) and the targeted viral genes (e.g., [10]) have been proposed. These were partially motivated by shortages of supplies for SARS-CoV-2 testing, i.e., a significant challenge in the early days of the pandemic [11,12,13,14].

The present study describes the setting up and evaluation of a comprehensive SARS-CoV-2 testing platform in times of rapid need for a solution despite impending shortages. It also reports the results from applying this platform to ~10,000 probands from a geographically circumscribed area in north-eastern Germany over a ~10-week period between 21 March and 31 May 2020. Our findings suggest that the reported concept may serve as a blueprint for entering the ‘New Normal’ during times of COVID-19, and for how to effectively fight similar outbreaks in the future.

## 2. Materials and Methods

### 2.1. Portal Description

A web application to be used on smartphones, tablets, and personal computers was developed as information technology support for our holistic SARS-CoV-2 testing approach. The registration process is based on the user’s email and a self-defined password. It fulfills all applicable requirements for the use of digital services, such as (i) specification and control of sufficiently secure passwords, (ii) protection by Captcha against machine access, and (iii) consent to the privacy policy and the general terms of use. After successful registration, the registrant receives an email asking for registration to be confirmed.

Information intake covers basic personal data (name, first name, date of birth, gender, address, email address, phone number). In a test-wise manner, users are also asked to indicate whether they are suffering from cough, fever, or other influenza-like symptoms, and whether they are aware of having been in contact with a confirmed COVID-19 patient. Following the granting of consent to the analysis and to data processing, the user receives an email with a PDF that confirms registration and consent, and contains a person-specific QR-code. Upon sample collection, this QR-code is presented digitally or in print. It is used to identify the person, and to link the test to the material ID, with which sample collection tubes are prelabeled. During sample processing in the lab, the portal captures all major steps. It also has an interface to receive the test result from the device applied for RT-PCR. After medical validation, report generation is initiated by the upload of all relevant data to the report form template. Users receive an email once their results are available in the Corona Test Portal. After log-in to the portal, reports can be viewed and downloaded in PDF format.

### 2.2. Sample Collection and Storage

For the collection of the probands’ samples from the oropharyngeal region, two types of dry swabs were used: a commercially available product (Isohelix^TM^ DNA/RNA Buccal Swab SK-1S; Cell Projects Ltd., Harrietsham, UK) and our newly developed CentoSwab™ (CENTOGENE AG, Rostock, Germany). CentoSwab™ is a CE-labeled medical class 1 device manufactured according to ISO13485 by Rowemed AG (Parchim, Germany) exclusively and under the guidance of CENTOGENE AG following joint development and validation for use in RT-PCR based SARS-CoV-2 diagnostic testing. Sampled swabs were transported and stored prior to RNA extraction at 2–8 °C according to WHO recommendations [15].

### 2.3. RNA Extraction

RNA extraction applied either of two distinct approaches. A commercial spin column-based system (*Quick*-DNA/RNA MagBead, Zymo Research Europe GmbH, Freiburg, Germany) was used according to the manufacturer’s instructions. As an *in-house*-developed alternative, a guanidinium isothiocyanate (GITC)-based RNA lysis method [16] was modified for use with dry sample collection swabs as follows: 100 µL of GITC RNA lysis buffer (6 M GITC, 2% Sarcosyl, 20 mM EDTA, 0.1% Antifoam, 50 mM Tris/Cl, pH 8.0), and 200 µL PBS were added to sampled swabs, followed by lysis on a shaker at room temperature (RT) for 5 min. Of this lysate, 200 µL were then mixed with 270 µL Isopropanol and 50 µL bead suspension (Sera-Mag™ Magnetic SpeedBeads™; 50-fold dilution of the commercially available stock solution in Tris/EDTA buffer (TE) of pH 8.0). Lysate-contained nucleic acids were bound to the beads by shaking (400 rpm) at RT for 5 min. After settling of beads on a magnetic rack for 15 min, the supernatant was discarded, and the beads were washed three times (first step: 150 µL Isopropanol; 2nd and 3rd step: 200 µL 80% (*v*/*v*) Ethanol), dried at RT for 5 min, and suspended in 30 µL TE.

### 2.4. RT-PCR and Result Evaluation

For the detection of viral RNA, a one-step RT-PCR system was used (LightMix^®^ SarbecoV E-gene plus EAV, TIB MOLBIOL, Berlin, Germany). The system consists of a TaqMan polymerase and a two-tiered approach carried out on a LightCycler^®^ 480 (Roche Diagnostics, Rotkreuz, Switzerland).

The experiments that aimed at defining sensitivity, as well as those comparing swabs and RNA extraction methods, were based on positive controls. These were prepared by spiking the swab loading fluid with an interlaboratory testing-derived high titer sample. Based on the dilution series, the concentration of the spike-in was chosen such as to result in an expected Cp value of 32. For defining the lower limit of detection, we prepared serial dilutions of positive reference material (positive control as contained in the RT-PCR system; see above), and considered the indicated minimum copy content. We then ran RT-PCRs in triplicate, and documented the observed Cp values. Taking into account the manufacturer’s recommendations that (i) a Cp value cutoff of 36 is to be used, and (ii) that this value should be 1–2 higher than the positive control, we defined the LLOD as the concentration at which all three Cp values are <34.

For proband testing, the presence of the *E* gene of SARS-CoV-2 was evaluated in a primary screening step and, if positive, the presence of the virus’ *RdRP* gene was evaluated in a secondary confirmation step. A minimum of two negative controls (water instead of RNA as a template) and of two positive controls (artificial viral RNA as provided along the RT-PCR kit) were run on each RT-PCR plate. Positive samples were defined by the Cp values for both *E* gene and the *RdRP* gene being <36.

### 2.5. Tested Individuals

The test has been applied on a local, national, and international level. While a specific cohort of ~500 symptomatic patients was tested early after the establishment of our assay, the majority of tests were not initiated by the presence of suggestive symptoms. Testing was offered to employees of companies, to personnel of critical infrastructure, to elderlies in nursing homes, and to interested individuals in a ‘walk-in’ setting. In the current study, we focus on 17,545 tests that were performed on 9720 individuals from the north-eastern part of Germany between 21 March and 31 May 2020.

### 2.6. Virus Genome Sequencing and Analysis

Double-stranded complementary DNA (cDNA) was obtained from isolated RNA by using a combination of commercial kits and primers (ProtoScript II First Strand cDNA Synthesis Kit; NEBNext Ultra II Nondirectional RNA Second Strand Synthesis Module; Random Primer 6; all New England Biolabs, Ipswich, MA, USA) according to the manufacturer’s instructions. Target capture of the viral cDNA was carried out with a targeted enrichment panel (SARS-CoV-2 Research Panel, Twist Bioscience, San Francisco, CA). Sequencing used NextSeq500 sequencers (Illumina, San Diego, CA, USA) to produce 2 × 150-bp reads. Raw sequencing data were converted to standard fastq format using bcl2fastq (Illumina). The adapter sequences were trimmed using the Trimmomatic 0.39 tool [17]. The DRAGEN RNA Pathogen Detection pipeline was used for alignment and variant calling [18]. Consensus FASTA files for four positive samples were created and deposited to the GISAID database on 8 June 2020. Phylogenetic analysis of the samples was performed as detailed previously, following the standard protocol for analysis of SARS-CoV-2 genomes as provided by Nextstrain [19].

## 3. Results

### 3.1. Corona Test Portal

A minimum valuable product (MVP) version of the Corona Test Portal was released initially; improvements and extensions are continuously being added (current version: RegistrationCENTOGENE N.V. (2020). Corona Test Portal (2.4.4) at http://corona.centogene.com). For support questions, less than 0.5 full-time equivalents are necessary, and we run without an outage and an available >99.5%. In the case there are no further actions resulting from a SARS-CoV-2 test, all personal data is automatically deleted after three months. On request, deletion is carried out in advance. The associated medical findings are stored in accordance with legal regulations. All data processing takes place exclusively in accordance with the consent of the users. The Corona Test Portal has been audited by Datenschutz cert GmbH and has been awarded the ips^®^ (internet privacy standard—a nationally recognized standard for data protection and IT security testing of web services) seal of approval.

### 3.2. General Validation of the Virus Detection Approach

In order to demonstrate the specificity of our assay, we used two types of negative controls: (i) swabs that were not loaded by oropharyngeal sampling (‘unsampled swabs’) as input for the RNA extraction (*n* = 35), and (ii) one-step RT-PCR with no template added (*n* = 30). We did not observe an amplification product for the *E* gene in any of the 65 reactions, resulting in a specificity of 100%. For defining sensitivity, we spiked 50 swabs with viral particles obtained from a high titer proband sample (thereby generating positive contrived samples). In 49 of the corresponding reactions, an *E*-gene-derived amplification product was obtained. Based on this finding, we estimate the assay’s sensitivity to be 98%. In a similar set of experiments based on between four and thirty independent reactions, intrarun precisions were found to be 1.8% (*E* gene) and 2.6% (*RdPR* gene), while interrun precisions were 1.9% (*E* gene) and 2.4% (*RdPR* gene). Table 1 summarizes the above-described validation experiments. Upon serial two-fold dilutions of the contrived positive proband sample, Cp values correlated with dilution steps over a wide range of concentrations (not shown). The lower limit of detection was determined as two viral particles per µL swab loading fluid.

### 3.3. Validation of an Alternative Sample Collection Swab and of an Alternative RNA Extraction Approach

While the cohort that is reported by the present study was screened using the above-described procedure, alternatives for sample collection and RNA extraction were established in parallel.

**Figure 1:**
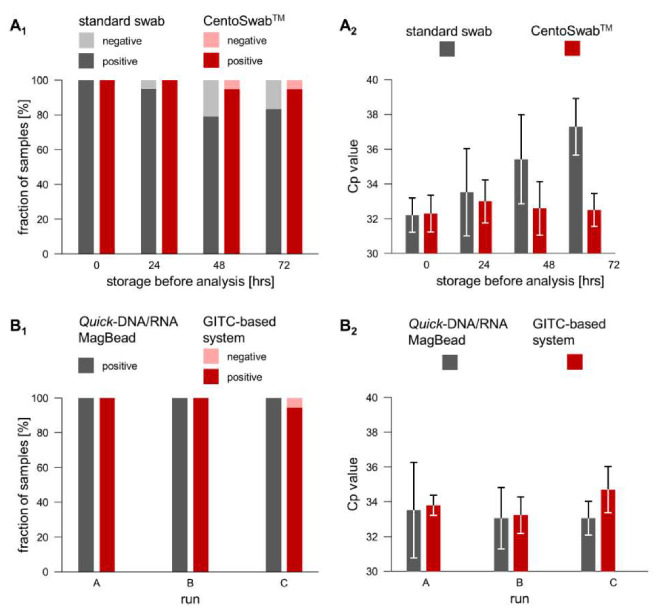
Comparative validation of options for sample collection and for RNA extraction. (**A**) RNA stability on a standard buccal swab vs. on CentoSwab^TM^ was compared regarding the fraction of samples testing positive (**A_1_**), and the number of RT-PCR cycles required to reach the signal threshold in RT-PCR (Cp value) (**A_2_**). (**B**) The ability to extract analyzable RNA was compared between a commercially available system and the in-house-developed regarding the GITC-based system in three independent runs (termed A, B, and C). Parameters used for comparison were the fraction of samples testing positive (**B_1_**), and the number of RT-PCR cycles required to reach the signal threshold in RT-PCR (Cp value) (**B_2_**).

A commercially available product (Isohelix^TM^ DNA/RNA Buccal Swab SK-1S; Cell Projects Ltd., Harrietsham, UK) was compared to the newly developed CentoSwab™ (CENTOGENE AG, Rostock, Germany). Unsampled swabs (*n* = 20 each as negative controls) did not give an amplification product after storage at 4 °C for up to 72 h. For swabs that had been spiked with high viral titer specimen, the fraction of reactions resulting in an amplification product decreased after prolonged storage; this issue was less pronounced for CentoSwab^TM^ (Figure 1A_1_). Similarly, an ever-increasing number of RT-PCR cycles was necessary to amplify from the commercial swab, but not from CentoSwab^TM^ (Figure 1A_2_). The coefficients of variation in these validation experiments ranged from 3.1% to 7.3% for the standard swab, and from 2.9% to 4.7% for CentoSwab^TM^.

RNA was either extracted by the spin column-based standard system or by the in-house-developed GITC-based system (compare Methods section). None of the negative controls (*n* = 20 unsampled swabs for each approach) resulted in an amplification product. From positive contrived specimen (swabs spiked with high viral titer; three independent runs with 6 to 18 independent experiments each), viral RNA was successfully amplified for 32 of 32 samples extracted by the standard system (100% sensitivity), and for 30 of 31 samples extracted by the GITC-based approach (96.8% sensitivity) (Figure 1B_1_). Mean corresponding Cp values were highly similar for both systems in all three runs (Figure 1B_2_); the associated coefficients of variation were slightly higher for the standard system (2.9% to 8.2% vs. 1.7% to 3.8%).

### 3.4. Description of the Tested Cohort

**Figure 2:**
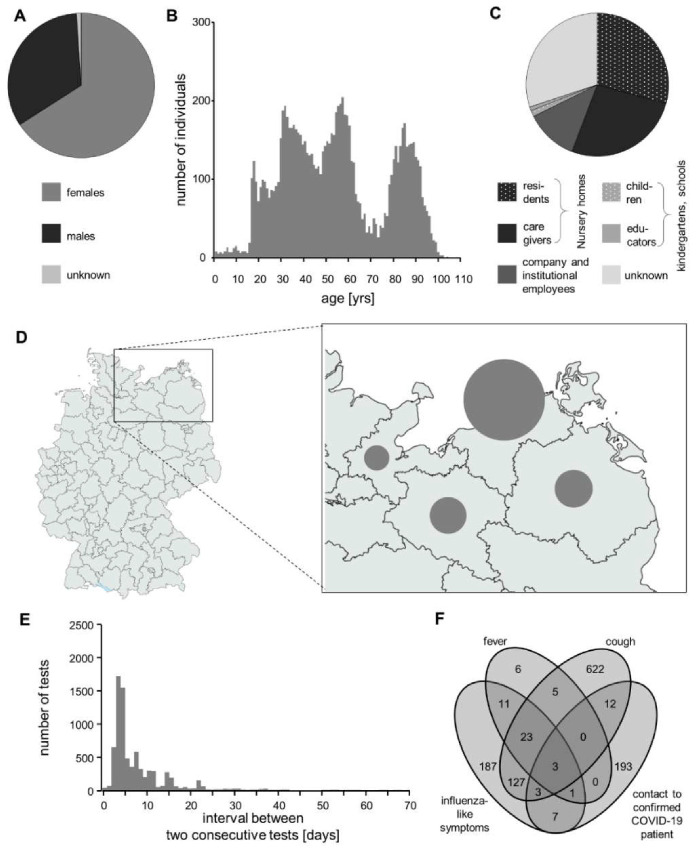
Cohort of tested individuals. (**A**) Stratification of individuals according to gender. (**B**) Distribution of ages at testing. (**C**) Stratification of individuals according to the background. (**D**) Geographical origin of the tested individuals. In the enlargement to the right, the relative size of the circles denotes the number of individuals from the corresponding area. (**E**) Distribution of intervals between tests upon recurrent testing. (**F**) Venn-diagram showing how often the indicated four items were associated with each other.

Of the 9618 tested individuals for which gender was available, 6401 were females (65.9%) and 3217 were males (33.1%) (Figure 2A). Age varied widely (Figure 2B). The largest group of individuals (*n* = 5428; 55.8% of all) was from nursery homes (Figure 2C). Tested individuals originated from different areas in north-eastern Germany (Figure 2D). While 7167 individuals (73.7% of all) got tested once, the remaining 2553 individuals (26.3%) got tested recurrently (from 2 to 22 times). The time interval between tests ranged widely, with three days and four days being outstandingly frequent (Figure 2E). Only a small fraction of individuals were reporting unspecific symptoms like fever (*n* = 49; 0.3% of all), cough (*n* = 796; 4.5%), or other influenza-like symptoms (*n* = 362; 2.1%); the same was true regarding contact with confirmed COVID-19 patients (*n* = 219; 1.2%). When these features were present, however, they tended to co-occur (Figure 2F).

### 3.5. Positively Tested Individuals

A total of 5 of the 9720 individuals tested positive (0.05%). Neither the male/female ratio nor the mean age of these positives differed from that in negatives. While three of the positive individuals did not self-report any potentially infection-related items, one reported to have had contact with a confirmed COVID-19 patient, and another one reported to suffer from cough and influenza-like symptoms at the time of sample collection. The SARS-CoV-2 status in all five positive samples, as well as in ten randomly selected negative samples, was confirmed by an external lab.

**Figure 3:**
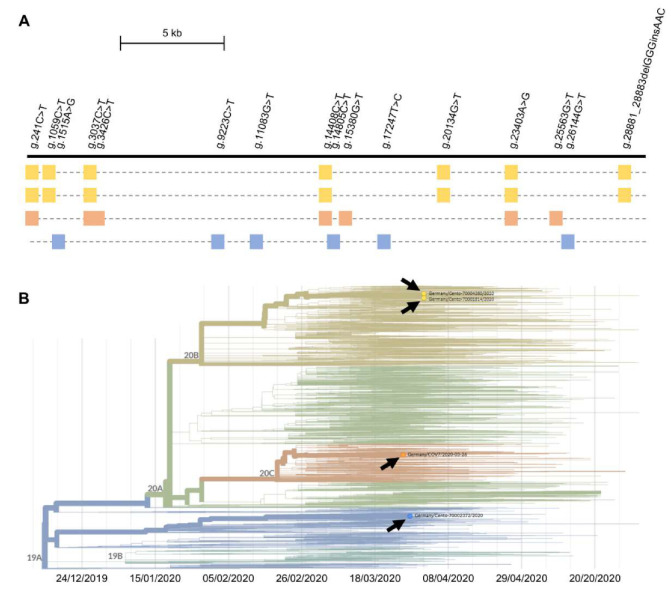
Genomes of the identified Severe Acute Respiratory Syndrome Coronavirus-2 (SARS-CoV-2) species. (**A**) To scale scheme of the 29,903 bp viral reference genome as a horizontal black line (NC_045512). Variants detected in at least one of the four sequenced specimens are indicated above; presence of the variant in question in a sample (samples as stippled horizontal lines) is indicated by a colored square. (**B**) Phylogenetic analysis of the four SARS-CoV-2 genomes (colors corresponding to those used in (A); image generated using tools as provided by Nextstrain [19]). 19A, 19B, 20A, 20B, and 20C denote the currently recognized five major clades; arrows indicate where the presently analyzed virus genomes map in the overall phylogenetic tree.

Reconstruction and phylogenetic analyses of the virus genomes from four of the positives revealed that the detected strains belonged to three of the five currently defined major clades (Figure 3).

## 4. Discussion

The COVID-19 pandemic hit unexpectedly and spread rapidly. The identification of infected individuals became imperative almost overnight. Testing laboratories were consequently faced with a variety of challenges at diverse levels. The present study describes the development of individual solutions that, in combination, represent a holistic package for a scalable, rapid, and high-quality SARS-CoV-2 testing. It also reports on the application of this package for preventive testing in a circumscribed geographical area.

Detection of the virus in proband samples is the central step of any testing procedure. While there is an increasing number of methodological approaches [20,21], reverse transcription of RNA followed by amplification of the resulting cDNA has been the primary strategy, and remains the most widely used [8,22]. We combined appropriate molecular biological components and validated the resulting diagnostic assay. Performance parameters were comparable to those of similar approaches [12,23]. Of note, sensitivity and specificity as the probably most critical parameters meet current FDA recommendations [24].

We decided to implement a back-up concept in order to be able to meet the challenges resulting from a shortage of commercially available test components. In relation to this, we took into account considerations on specifications for buccal swabs [25], and developed CentoSwab^TM^ as a new dry swab for oropharyngeal sampling. Validation of this tool against a commercial alternative showed similar or even better performance, especially regarding the long-term stability of sampled RNA (Figure 1A). In addition, we established an alternative RNA extraction method that is based on a classical protocol applying GITC [16]. When validating this method against the spin column-based standard RNA extraction, we found performance to be highly comparable (Figure 1B). Of note, similar protocols have been established by other laboratories in parallel [26]. While the initial shortages of supplies have largely been overcome, at least in the European Union, they can be expected to re-emerge in subsequent waves of COVID-19 and in future unrelated pandemics. Our pertinent back-up strategy exemplifies how potential shortages can be addressed despite a need to establish a testing platform in a very short time. Finally, we also developed a specific IT-solution that is independent of our laboratory information management system. It enables mobile-phone-application-based self-registration, and provides probands with a link to the test results once these are medically validated. As neither input nor output thus require skilled staff, and as a built-in interface automatically receives the laboratory results, this IT-solution further reduces turnaround time. The implementation of several alternatives to commercial solutions adds significant flexibility as well as the opportunity for rapid on-demand upscaling to our holistic testing platform.

We offered our testing platform in a primarily unbiased manner during the period covered by the present study. The eventual composition of the cohort (see Figure 2A–C for basic summary statistics) can still be expected to be influenced by numerous factors that impact on male/female ratio and age distribution. Similarly, the peaks at days three and four in the test interval data for recurrent testing (Figure 2E) likely mirror attempts to get tested twice per week. The observed overall positivity rate of 0.05% in our cohort is below that reported by most other screening studies, even when compared to national levels of mildly hit countries [27]. A potential concern about false negativity can be rejected based on our test validation results, and on the fact that a positivity rate of >3% was observed in an independent cohort of symptomatic individuals tested in parallel (data not shown). Still, and as is characteristic for COVID-19, very early phases of the infection can be expected to be diagnosed with lower sensitivity [28]. General concerns about too low specificity (i.e., significant false positivity) [29] can also be rejected based on our low positivity rate. The low number of positives in our cohort is likely explained by the Mecklenburg Western-Pomerania area generally being the least affected by COVID-19 in Germany [30], not mutually exclusive explanation is the explicit inclusion of asymptomatic individuals. Less than 5% of all probands did, in fact, self-report a potentially COVID-19-related observation. With one exception, even the positive cases reported no symptoms. This is consistent with the overall high frequency of asymptomatic individuals infected with SARS-CoV-2 [31]. We believe that this focus on asymptomatic individuals, together with recurrent testing, represents a valid strategy for locally keeping COVID-19 under control.

Interestingly, virus genome sequencing in positive individuals (Figure 3) revealed that distinct major virus clades [32] hit the local area. Two strains represent the early waves from the far East to Europe, while the third was a strain that was first seen in North America before ‘traveling back’ to Europe from there [33]. This is remarkable since SARS-CoV-2 obviously hits even remote regions recurrently. The amazing diversity in entry-points further emphasizes the need for a sustained preventive strategy.

In summary, we present a holistic SARS-CoV-2 testing approach that maximizes quality and flexibility. Its development, together with the reported application to mainly asymptomatic individuals, may serve as a blueprint for successfully entering the ‘New Normal’.

## Figures and Tables

**Table 1:** Basic validation characteristics of the assay. n.d., not done.

	Specificity	Sensitivity	Precision	
			Intrarun	Interrun
*E* gene	65/65 (=100%)	49/50 (=98%)	1.8%	1.9%
*RdRP* gene	n.d.	n.d.	2.6%	2.4%
